# The Influence of Different Cholecalciferol Supplementation Regimes on 25(OH) Cholecalciferol, Calcium and Parathyroid Hormone after Bariatric Surgery

**DOI:** 10.3390/medicina55060252

**Published:** 2019-06-06

**Authors:** Hendrika J.M. Smelt, Sjaak Pouwels, Johannes F. Smulders

**Affiliations:** 1 Department of Nutrition, Catharina Hospital, 5623 EJ Eindhoven, The Netherlands; marieke.smelt@catharinaziekenhuis.nl; 2 Obesity Center, Catharina Hospital, 5623 EJ Eindhoven, The Netherlands; frans.smulders@catharinaziekenhuis.nl; 3 Department of Surgery, Haaglanden Medical Center, 2512 VA The Hague, The Netherlands; 4 Department of Surgery, Catharina Hospital, 5623 EJ Eindhoven, The Netherlands

**Keywords:** vitamin D supplementation, cholecalciferol, parathyroid hormone, serum vitamin D, bariatric surgery

## Abstract

*Background and objectives:* Vitamin D is an essential vitamin that plays a key role in maintaining physiological calcium balance, and is also a pivotal element in the formation of bone structure. Vitamin D deficiency is associated with a wide array of clinical symptoms. Vitamin and mineral deficiencies are quite common prior to and after bariatric surgery, and therefore we have evaluated the effects of two different cholecalciferol supplementation regimes on serum calcium, 25(OH) cholecalciferol, and parathyroid hormone (PTH). *Materials and Methods:* In this retrospective matched cohort study, two different cholecalciferol supplementation regimes were compared. Group A consisted of 50 patients who had 1000 mg calcium and 800 IU cholecalciferol. In Group B, 50 patients had 1000 mg calcium and 800 IU cholecalciferol with an additional 1 mL liquid cholecalciferol (50,000 IU) monthly. The primary outcome was the effects on blood serum levels of calcium, 25(OH) cholecalciferol, and PTH. *Results:* In group A and group B, there were significant increases in 25(OH) cholecalciferol, with a higher delta in favor of group B (for all three *p* < 0.001). A decrease was seen in PTH (*p* < 0.001), and no differences were measured in calcium levels in both groups. *Conclusion:* Our study suggests that an additional 1 mL cholecalciferol (50,000 IU) monthly can result in less biochemically 25(OH) cholecalciferol deficient patients after bariatric surgery. No effects were seen on the calcium balance. However, larger randomized clinical trials need to be done to assess the effects on clinical outcomes like bone health and fracture risk.

## 1. Introduction

In general, vitamin D is part of the complex physiology maintaining calcium balance and bone structure, but also aids in the absorption of calcium from the intestine and reabsorption in the distal renal tubules (in presence of parathyroid hormone (PTH)). Deficiencies in vitamin D can increase the risk of osteoporosis and fractures, but also have effects on the immune system and muscle strength [1]. A vitamin D deficiency is often accompanied by a reduced dietary calcium intake, but this does not necessarily affect the serum calcium [1,2]. Physiologically, when there is a fluctuation in calcium levels, PTH levels will also fluctuate. In case of a decreased blood calcium level, PTH will increase [1,2]. Clinical symptoms of a hypocalcaemia can vary, but symptoms like leg cramping, tetany, and neuromuscular hyper excitability are often reported [2].

Vitamin D deficiencies are common in bariatric surgical practice, and the reported prevalence prior to surgery varies between 54% and 80% [3]. In patients with obesity, this is often a combination of several problems: (1) Limited sun exposure, (2) decreased bioavailability of vitamin D due to sequestration in the excess fatty tissue, and (3) inadequate dietary intake of vitamins and minerals [3]. A secondary hyperparathyroidism can also contribute negatively because it results in increased 25(OH)D hydroxylation, therefore decreasing vitamin D [2,3]. In addition to hyperparathyroidism, several cases of osteomalacia have been described following bariatric surgery [4,5]. It needs to be taken into account that these are rare phenomena. 

Currently, it is advised by several guidelines that blood levels of vitamin D of ≥75–80 nmol/L are optimal, especially for patients before and after bariatric surgery [6,7]. However, if deficiencies occur, there is no consensus on how we should supplement these patients to achieve normal vitamin D levels. In our bariatric practice, we have used several supplementation regimes. One of them was 1000 mg oral calcium carbonate and 800 IU oral cholecalciferol daily from three weeks postoperatively. Unfortunately, too many patients remained deficient after this regimen, and therefore we added additional cholecalciferol to our supplementation protocol.

However, it is unclear whether this switch has effects on 25(OH) cholecalciferol, PTH, and calcium. We aimed to evaluate the effects of these supplementation regimes on blood levels of calcium, 25(OH) cholecalciferol, and PTH. Therefore, we hypothesize that a supplementation regime of 800 IU oral and additional cholecalciferol monthly and lifelong leads to higher 25(OH) cholecalciferol levels and less deficiencies.

## 2. Materials and Methods

### 2.1. Patient Selection

In this pilot study, we included 100 patients who have had bariatric surgery in the period of June 2015 until January 2016. All patients underwent a sleeve gastrectomy or Roux-en-Y gastric bypass either as a primary or a revisional procedure in the Obesity Center Catharina Hospital Eindhoven, the Netherlands. Patients with kidney disease or gastrointestinal disorders suggestive for malabsorption were excluded from this study.

The included patients from group A (*n* = 50) were selected on the operation date in June 2015 and started with our standard supplementation regime of 1000 mg oral calcium carbonate and 800 IU oral cholecalciferol. The included patients from group B (*n* = 50) were selected on the operation date in January 2016 and used the previously mentioned supplementation regime and additional 50,000 IU liquid cholecalciferol monthly. Both groups were matched for age, gender, preoperative body mass index (BMI), current BMI, and surgical procedure. Due to the retrospective nature of this study, approval from the Institutional Review Board of the Catharina Hospital Eindhoven was not needed.

### 2.2. Blood Sampling and Definitions

Baseline blood analysis of all patients was done three months preoperatively and six months postoperatively. For this study, serum calcium, vitamin D, and PTH were assessed. Vitamin D deficiency was defined as serum vitamin D < 75 nmol/L. Vitamin D (25-hydroxy vitamin D) was determined in serum by an immunometric competition assay on Liason^®^ using Diasorin^®^ reagents. Reference values of calcium and PTH were 2.10–2.55 mmol/L and 1.6–6.9 pmol/L, respectively.

### 2.3. Correction of Deficiencies Preoperatively

Preoperative deficiencies in all patients was treated with cholecalciferol. Dosage: 50,000 IU/week for the first six weeks, and afterwards 50,000 IU monthly up to the bariatric procedure.

### 2.4. Statistical Analysis

Data were retrospectively collected, managed, and analyzed using SPSS version 22, for Windows (SPSS Inc., IBM Corporation, Armonk, NY, USA). Quantitative data are denoted as mean ± standard deviation (SD). Categorical variables were presented as frequencies with percentages. The χ-square test was used to compare categorical variables among groups. Distribution of the data was verified using the Shapiro–Wilks test. Depending on the distribution, the paired *t*-test was used for parametric data and the Mann–Whitney U test for non-parametric data. To analyze differences in calcium, 25(OH) cholecalciferol, and PTH levels, the deltas were calculated (follow-up levels minus baseline levels), and these values were compared between groups using the one-way analysis of variance (ANOVA) test. Deltas were determined by the following calculation: Follow-up levels minus baseline levels. A *p*-value ≤ 0.05 was considered statistically significant. Summative figures and tables were used where necessary.

## 3. Results

This work has been presented at the IFSO World Congress in Dubai and the meeting abstract was published in Obesity Surgery [8].

Table 1 gives an overview of the baseline characteristics of the patients included in each group. None of the included patients had symptoms of hypocalcaemia. In case of a Roux-en-Y Gastric Bypass (RYGB), a Roux limb of 150 cm was used.

### 3.1. 25(OH) Cholecalciferol

Both groups showed increases in 25(OH) cholecalciferol levels in the follow-up (*p* < 0.001 for groups A and B) (Table 2). Of all included patients, 91 patients had a 25(OH) cholecalciferol deficiency (<75 nmol/L) preoperatively. In group A, 47 patients had a 25(OH) cholecalciferol deficiency at baseline, and 35 had it in the follow-up. In group B, 44 patients had a 25(OH) cholecalciferol deficiency at baseline, and 10 had it in the follow-up.

### 3.2. Calcium and Parathyroid Hormone Levels

No differences were seen in serum calcium in group A (*p* = 0.058) and group B (*p* = 0.930). Decreases in PTH were seen in the follow-up in group A (*p* < 0.032) and group B (*p* < 0.001) (Table 2).

### 3.3. Delta (Δ) of 25(OH) cholecalciferol, PTH, and Calcium between Both Groups

A significant difference in delta 25(OH) cholecalciferol (*p* < 0.01) was seen between group A (28.9 ± 16.7) and group B (47.2 ± 24.3), in favour of group B. Delta PTH in group A was –1.0 ± –0.3, and in group B it was –1.6 ± –1.1, but this difference was not significant (*p* = 0.336). No significant difference was seen in Δ calcium (*p* = 0.185). 

### 3.4. Sub-Analysis Between Sleeve Gastrectomy and Roux-En-Y Gastric Bypass

In group A, no significance differences were found in 25(OH) cholecalciferol (*p* = 0.573), calcium (*p* = 0.341), or PTH (*p* = 0.995) preoperatively. No significance differences were found in 25(OH) cholecalciferol (*p* = 0.851), calcium (*p* = 0.080), or PTH (*p* = 0.482) between laparoscopic sleeve gastrectomy (LSG) and RYGB patients six months postoperatively. In group B, no significance differences were found in 25(OH) cholecalciferol (*p* = 0.970), calcium (*p* = 0.796), or PTH (*p* = 0.127) preoperatively. No significance differences were found in 25(OH) cholecalciferol (*p* = 0.984), calcium (*p* = 0.615), or PTH (*p* = 0.992) between LSG and RYGB patients six months postoperatively.

## 4. Discussion

A standard supplementation regime of 800 IU oral cholecalciferol and 50,000 IU liquid cholecalciferol monthly leads to less deficiencies and results in a faster decline of PTH. There is no consensus about the right dose of vitamin D supplementation after bariatric surgery [9,10]. The recommended dosage of elemental calcium ranges from 1200 to 2000 mg daily [2,11,12]. Supplementation with 400–800 IU of vitamin D might not provide adequate protection for postoperative patients against an increase in PTH and bone resorption [13,14]. In the study of Flores et al., 56% of the bariatric patients had vitamin D levels of >75 nmol/L one year postoperatively by using 2000 IU vitamin D /day [15]. The study of Goldner et al. describes vitamin D levels of >75nmol/L in 44% of the patients with 800 IU vitamin D/day and in 78% of the patients with 2000 IU vitamin D/day [16,17]. 

In our study, the cholecalciferol supplementation regimen with 800 IU cholecalciferol was simply not enough to biochemically restore 25(OH) cholecalciferol levels in 35 of the 50 patients. The standard supplementation regimen with additional 50,000 IU cholecalciferol monthly showed that 10 of the 50 patients were still having a deficiency. A higher cholecalciferol dosage gives a faster decline of PTH and may result in differences in absorption. The high-dose cholecalciferol regime gives better results (in terms of a more prominent decrease in patients with a cholecalciferol deficiency). However, there are still a small proportion of patients that remain deficient, despite the high doses of cholecalciferol. This might be related to compliance of intake of the supplements. This is the most difficult point in this study because this is unfortunately difficult to control. 

Next to the above-mentioned factors, we need to take procedure-specific influences into account, because bariatric procedures are not all the same regarding absorption of vitamins and minerals, especially calcium and vitamin D [7,9]. Calcium is mainly absorbed in the duodenum and proximal jejunum, which is facilitated by the presence of vitamin D in an acidic environment [9]. Vitamin D, on the other hand, is absorbed in the distal jejunum and the ileum. As mal- or hypo-absorptive effects increase, so does the hypo-absorption of vitamins in specific parts of the small intestine [2].

However, in this study, no significant differences in 25(OH) cholecalciferol, calcium, and PTH were found between the different surgical procedures.

## 5. Limitations

Only biochemical outcomes were presented in this study. The clinical outcomes were not mentioned. A larger and better-designed study including DEXA scans is necessary to understanding the effects on calcium metabolism and bone density. 

The effect of calcium supplementation was not included in this study. However, there is no clear policy for calcium supplementation and no large randomized trials that confirm the optimal regime after bariatric surgery [9]. Finally, there is a known seasonal variability regarding (metabolites of) vitamin D, and it is therefore difficult to correct for this statistically. Furthermore, to determine clinical outcomes (osteoporosis risk and fracture incidence), a minimum follow-up of a few years is needed.

## 6. Conclusions

According to this pilot study, a standard daily supplementation regime of 800 IU orally and additional liquid cholecalciferol of 50,000 IU monthly results in higher serum 25(OH) cholecalciferol and less deficiencies. This regimen cannot treat all vitamin D deficiencies, and therefore, lifelong follow-up is necessary. A randomized clinical trial is necessary to investigate different vitamin D supplementation regimens, the influence on PTH, and the influence of calcium supplementation after bariatric surgery.

## Figures and Tables

**Table 1:** Baseline characteristics of the included patients (*n* = 100) (mean ± standard deviation (SD)).

Different Groups	Group A (*n* = 50)	Group B (*n* = 50)	*p* Value
Age (years)	43.8 ± 11.6	47.5 ± 9.7	*p* = 0.075
Gender (*n*)			
- Male:female	9:41	10:40	*p* = 0.799
Preoperative body mass index (kg/m²)	42.6 ± 5.7	42.5 ± 5.2	*p* = 0.911
Current body mass index (kg/m²)	31.8 ± 4.6	31.7 ± 4.6	*p* = 0.879
Procedures (*n*)			*p* = 0.359
- SG	28	29	
- RYGB	18	20	
- Revision surgery	4	1	

SG = Sleeve gastrectomy, RYGB = Roux-en-Y gastric bypass.

**Table 2:** An overview of the baseline and postoperative levels of 25(OH) cholecalciferol, parathyroid hormone (PTH), and calcium levels per group (mean ± SD).

	Baseline Levels	Levels Six Months Postoperatively	*p* Value
Group A			
- 25(OH) cholecalciferol	37.8 ± 20.6	66.7 ± 18.5	*p* < 0.001
- PTH	7.5 ± 3.1	6.5 ± 2.9	*p* = 0.032
- Calcium	2.37 ± 0.08	2.39 ± 0.08	*p* = 0.058
Group B			
- vitamin D	47.0 ± 21.5	94.2 ± 25.7	*p* < 0.001
- PTH	6.8 ± 2.7	5.2 ± 1.6	*p* < 0.001
- Calcium	2.38 ± 0.11	2.38 ± 0.09	*p* = 0.930
